# Yoga in school sports improves functioning of autonomic nervous system in young adults: A non-randomized controlled pilot study

**DOI:** 10.1371/journal.pone.0231299

**Published:** 2020-04-13

**Authors:** Julia Frank, Georg Seifert, Rico Schroeder, Bernd Gruhn, Wiebke Stritter, Michael Jeitler, Nico Steckhan, Christian S. Kessler, Andreas Michalsen, Andreas Voss

**Affiliations:** 1 Department of Pediatrics, Jena University Hospital, Jena, Germany; 2 Department of Pediatrics, Division of Oncology and Hematology, Charité - Universitätsmedizin, Berlin, Germany; 3 Institute of Innovative Health Technologies IGHT, Ernst-Abbe-Hochschule Jena, Jena, Germany; 4 Institute for Social Medicine, Epidemiology and Health Economics, Charité - Universitätsmedizin, Berlin, Germany; 5 Department of Internal and Integrative Medicine, Immanuel Hospital, Berlin, Germany; University of Bern, SWITZERLAND

## Abstract

**Background:**

Yoga in school is a beneficial tool to promote the good health and well-being of students by changing the way they react to stress. The positive effects of yoga—taught in schools—on children, youth and young adults have been demonstrated in former studies using mostly subjective psychometric data.

**Aim:**

The present trial aims to evaluate the potential effects of yoga on autonomic regulation in young adults by analyzing heart rate variability (HRV).

**Methods:**

This study is a non-randomized, explorative, two-arm-pilot study with an active control group. Fourteen healthy young adults took part in a 10-week yoga program (90 min once a week) in school and were compared to a control group of 11 students who participated in conventional school sports (90 min once a week over 10 weeks). 24-hour electrocardiograms (ECGs) were recorded at baseline and following the 10-week intervention. From 20-minute of nocturnal sleep phases, HRV parameters were calculated from linear (time and frequency domain) and nonlinear dynamics (such as symbolic dynamics and Poincaré plot analysis). Analyses of variance (ANOVA) followed by t-tests as post-hoc tests estimating both statistical significance and effect size were used to compare pre-post-intervention for the two groups.

**Results:**

The statistical analysis of the interaction effects did not reveal a significant group and time interaction for the individual nocturnal HRV indices. Almost all indices revealed medium and large effects regarding the time main effects. The changes in the HRV indices following the intervention were more dramatic for the yoga group than for the control group which is reflected in predominantly higher significances and stronger effect sizes in the yoga group.

**Conclusion:**

In this explorative pilot trial, an increase of HRV (more parasympathetic dominance and overall higher HRV) after ten weeks of yoga in school in comparison to regular school sports was demonstrated, showing an improved self-regulation of the autonomic nervous system.

## 1. Background

Children, youth and young adults are regularly subjected to stress-inducing conditions and factors in school [1]. Stress is associated with a broad range of health and social problems [2] and has a negative effect on the ability to concentrate [3] leading among other influences to a poorer performance in school [4]. When subjected to stress, young people are more likely to develop unhealthy lifestyle habits, for example an unhealthy diet or drug use [5]. In an American study from the year 2011 the prevalence of psychological problems among 21 year-olds exceeded 80% [6], whereby most of these problems originated in childhood or adolescence [7].

Therefore, it is increasingly important for schools to convey to students skills and priorities that do not just improve the acquisition of theoretical knowledge and optimize performance. Schools should begin early on to promote good health and well-being by supporting activities that help students to combat stress and constructively react to psychological and social challenges. In accordance with the idea of “educating the whole child” [8], integrating mind-body methods such as yoga into the educational system has the potential to shift focus to whole-person approach.

Modern yoga, as it is generally taught and practiced in western society, aims to improve the functioning and interaction of body and mind. Physical exercises are combined with phases of deep relaxation and specific breathing and meditation exercises. Yoga serves to improve awareness of one’s own body, to calm the mind, to increase mindfulness and to improve physical fitness, among other effects [9]. As a result, physical and emotional reactions to stress and the perception of stress can be altered and healthier behaviors established [10]. Yoga can be practiced by almost anyone, independent of physical condition, because emphasis is placed on individual capabilities and not on competition [11]. For this reason, and because it requires so little equipment, the practice of yoga is well suited to school situations.

The broad range of positive effects of yoga on school students has been demonstrated in several studies. The most pronounced effects have been verified in the psychological realm, for example in relation to tension, anxiety, self-esteem and other mood indicators, and in relation to cognitive functions like memory [12]. Another review on the subject identified positive results especially in relation to such factors as emotional stability, attention control, cognitive efficiency, anxiety, negative thought patterns and both emotional and physiological excitability [13]. After a 12-week yoga intervention students felt more balanced and reported less negative behavior relative to stress factors [14]. Yoga has a positive influence on the emotional regulation of adolescents, who are particularly susceptible to emotional instability [15]. The manner in which adolescents deal with stressors emotionally has clear implications for the risk of developing psychological problems [16].

The studies cited primarily served to generate subjective psychometric data by such means as questionnaires, self-reports by the subjects and psychological and cognitive tests. However, more objective measurements of the effects of yoga interventions on school students are needed. Butzer et al. 2015 conducted an initial study in this direction, as they measured cortisol levels in the saliva of primary school students following a 10-week yoga intervention. They discovered that the baseline cortisol levels in seven to eight year-old children were significantly lower after the intervention [17]. A study also published in 2015 by Nagendra et al. investigated the effects of yoga on the cognitive ability, autonomic nervous system and heart rate variability (HRV)—measured by means of electroencephalography (EEG) and echocardiography (ECG) recordings—in engineering students. They found significant improvements in several cognitive functions—including memory, neuronal activity and alertness—and alterations in HRV tending towards parasympathetic dominance [18].

Heart rate variability is applied as an objective, non-invasive tool describing the influence of the autonomic nervous system on heart activity. It reflects the degree of the body’s physiological, endocrinal and emotional balance [19]. Chronic stress leads to dominance of the sympathetic nervous system and a decrease in vagal activity expressed by a lower HRV, for example reflected by a decreased energy spectral density in the high frequency range (HF)[20].

Some studies have demonstrated influences of yoga on the HRV in adults [21], though the evidence of the impact of yoga on HRV was judged by another review to be inconclusive [22]. HRV has scarcely been used as an objective parameter for the evaluation of yoga interventions in schools. The study presented here uses indices of HRV—in addition to psychometric and qualitative data that are published in another article [23]—to evaluate the effects of practicing yoga in school in young adults. For the investigation of HRV, both linear and nonlinear analytical methods (and their indices) are investigated, since regulation of heart rate is controlled or influenced by the activity of a variety of complex regulatory subsystems, such as vasomotor and respiratory centers, baroreflex and chemoreflex closed loop regulation, cardiovascular reflexes mediated by vagal and sympathetic afferences, and vascular- and thermo-regulation [24, 25].

## 2. Objective

The present trial aims to evaluate the potential effects of yoga on the autonomic regulation applying both linear and nonlinear short-term HRV analyses in young adults participating in a 10-week yoga course (one session per week of approximately 90 minutes) in comparison to conventional school sports.

## 3. Methods

### 3.1. Study design

This explorative pilot trial is a non-randomized, two-arm-study with an active control group: conventional school sports. The study protocol was approved by the ethics committee of the Charité-Universitätsmedizin Berlin on 5 April 2016. Written informed consent was obtained from the child's guardian and, if applicable, also from the adolescent in accordance with the Declaration of Helsinki. The trial was registered at Clinical Trials on 18 April 2016 (registration number: NCT02741973).We did the registration after the enrolment of the participants started because we had troubles with our account at the website (ClinicalTrials.gov). The authors confirm that all ongoing and related trials for this intervention are registered.

Participants were recruited in March 2016 from two secondary schools in Berlin (German: Oberstufenzentren). At these institutions, students can obtain both a general secondary school qualification and a complete professional training.

Three sports teachers each selected one of their classes to participate in the yoga exercises and another class to act as a control group in accordance with comparability criteria. Six classes were thus included in the study. The three yoga intervention classes were instructed by external yoga teachers. The three control groups participated in their regular sports class but not in yoga courses.

**Fig 1:**
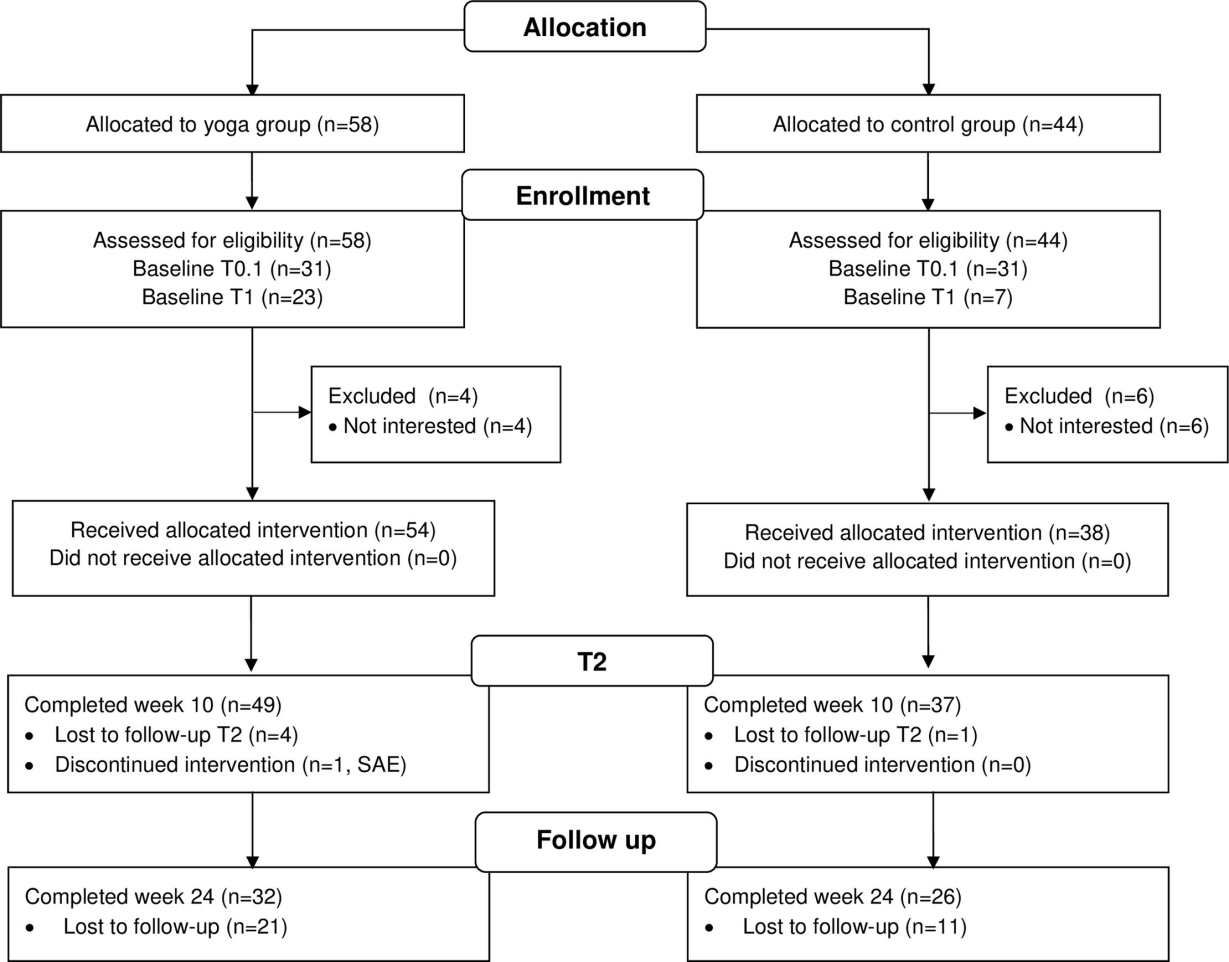
Flow diagram. It displays the progress of all participants through the trial.

A flow diagram of the study is presented in Fig 1.

**Fig 2:**
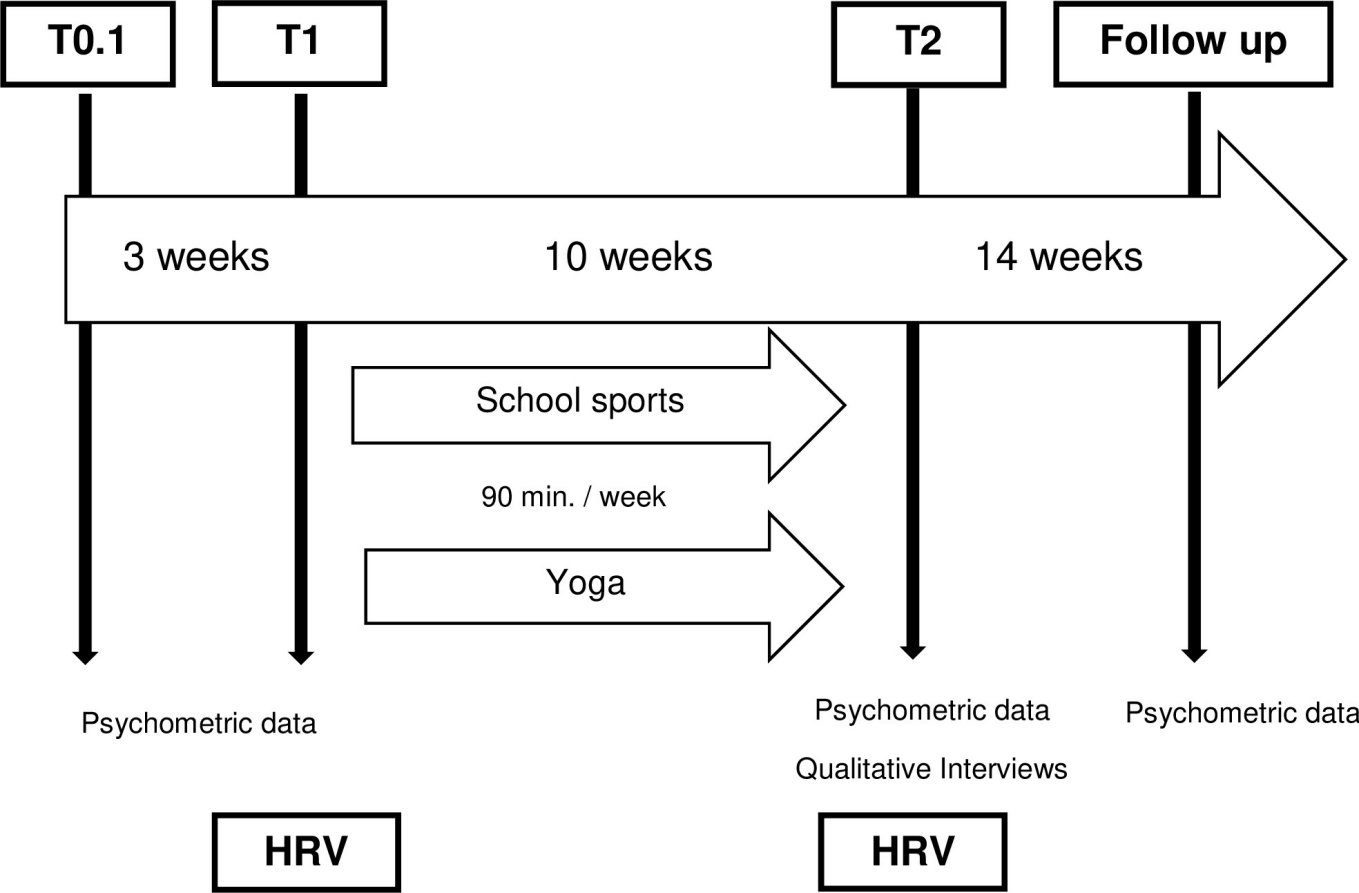
Study design. Timelines: T0.1—start of the study and first collection of psychometric data, T1—first ECG recording before intervention, T2—second ECG recording and collection of psychometric data following immediately the intervention.

Psychometric data were collected before the intervention, in March 2016, immediately following the intervention, after ten weeks, and six months later. In addition, after a period of ten weeks focus group interviews of approximately 45 minutes were conducted (Fig 2). These results are presented in a separate publication [23].

For the present analysis of HRV, a subgroup of 34 school students was willing to submit to 24-hour ECG monitoring in order to measure stress reaction prior to (T1 in Fig 2) and following the 10-week intervention (T2 in Fig 2). In appreciation of their participation, each of the students who underwent the long-term ECG received a gift certificate.

### 3.2. Participants

Subjects were included in the study if they fulfilled both of the following conditions: they 1) were secondary school students and 2) provided informed consent. Students were excluded from the study if they 1) suffered from a severe chronic or acute disease, 2) were pregnant, 3) were not mobile or suited for gymnastic exercises due to orthopedic, neurological, mental or other medical conditions or 4) had participated in any other trial. Each participant received an explicit identification number.

A total of 102 subjects were screened for eligibility and 92 students (53% female) between the ages of 17 and 29 years (mean age 19.6±2.22 years) were enrolled in the study in March 2016 (Fig 1). Of these study participants, 54 young adults took part in three yoga classes, while 38 young adults participated in three sports classes (controls). Each class had between 12 and 20 participants.

Of the 92 study participants 34 young adults agreed to the ECG recordings and underwent this procedure. Fourteen of the 25 evaluable ECG data sets originate from the yoga group (57% female) and 11 from the control group (46% female) (Table 1). The remaining nine ECG recordings contained too many artefacts (e.g. movements, loose lead and electromagnetic interference (EMI) artefacts) and could not be analyzed for this reason. The ages of the subjects ranged from 17 to 25 years (mean age 19.9±4.04 years).

**Table 1:** Study population characteristics.

	*Yoga group (n = 14)*	*Control group (n = 11)*
*Female*	57.1%	45.5%
*Age (years)*	19.7 ± 1.9*	20.1 ± 2.1*
*BMI (kg/m^2^)*	23.66 ± 4.03*	22.47 ± 1.91*

n—number of participants

*—mean ± standard deviation

BMI—body mass index

### 3.3 Interventions

Over a period of ten weeks, once a week, either a yoga session for the yoga group or in parallel to that a sports lesson for the control group was carried out for about 90 minutes. The courses took place in the framework of the routine sports class in the school’s gymnasium.

#### 3.3.1. Yoga

The yoga sessions were conducted by three teachers (two men and one woman), one for each class. All of them are certified by a professional association of yoga teachers in Germany (“Berufsverband der Yogalehrenden in Deutschland e.V.”–BDY) and had been trained in yoga for over 20 years. A standard operating procedure (SOP) was developed in order to ensure that the three yoga classes were comparable in their structure and implementation. The yoga sessions were attended by the regular school sports teachers.

The yoga intervention was conducted in an ideologically neutral manner. It included the four standard basic elements of western yoga: (1) physical postures (in Sanskrit *asana*), (2) breathing exercises (*pranayama*), (3) relaxation techniques (for example *shavasana)* and (4) meditation.

Each yoga class was structured as follows: 1) welcome and feedback on the last session (5 minutes), 2) introduction and awareness exercises (10 min.), 3) warm-up (10 min.), 4) more intensive *asana* practice (20 min.), 5) floor exercises and transition to relaxation (15 min.), 6) light *pranayama* sitting exercises (5 min.), 7) silent meditation while sitting (5 min.), and 8) feedback (5 min.).

The aim of the first phase of each yoga class was to strengthen the body and focus the mind. In the second part calm postures for limb lengthening and regeneration were practiced. Focus was then placed on breathing exercises and meditation before practicing the final relaxation posture known as *shavasana*. The students were also encouraged to practice yoga at home.

#### 3.3.2. School sports

In the control group, the students were taught by their regular sports teachers in accordance with the standard curriculum, whereby emphasis was placed on sports like basketball, volleyball and badminton. These students did not participate in the yoga class but were given a voucher that would allow them to take ten comparable yoga lessons outside of school following the conclusion of the study. The participants agreed not to significantly modify their routine activities or to begin any other exercise or stress reduction programs during the study period.

### 3.4. Outcome parameters: Heart rate variability

Two-lead Holter ECGs were recorded using the ePatch AMS3000 from DELTA, Hoersholm, Denmark and digitized with a sampling rate of 512 Hz and a resolution of 12 bits. From the two raw 24h Holter ECG recordings for each subject, two time series of 20-minute beat-to-beat intervals (BBI) from nocturnal sleep phases were extracted using Cardiscope™ (HASIBA Medican GmbH supplied by SMART medical) and an in-house stationary detection Matlab software: (T1) was taken prior to the yoga intervention as a baseline and (T2) was taken shortly after the 10-week intervention (Fig 2). For the selection of the 20-minute sleep phases, four-hour BBI sleep phases, which appeared largely free of external disturbances, were initially selected manually from the 24-hour ECGs (by means of Cardiscope^TM^) depending on the individual sleep time of the subjects. To ensure reliable HRV measurements from these four-hour BBI sleep phases, only 20-minute periods during nocturnal non-rapid eye movement sleep that met the pre-selection criterion for most stationary segments were automatically extracted and visually after-checked by means of a stationary detection algorithm (Matlab). The algorithm for extracting stationary segments can be found in [26].

Occasionally occurring ectopic beats and disturbances or artefacts were interpolated in the given BBI series applying an adaptive filter [27] to generate normal-to-normal (NN) interval time series. Afterward, following linear and nonlinear HRV analysis methods were used to calculate various HRV quantifying indices.

#### 3.4.1. Linear methods

Following linear HRV standard indices from the time and frequency domain were calculated as suggested by the guidelines of the Task Force of the European Society of Cardiology [28] and Voss et al. [25]. Based on the NN interval time series, from time domain (TD) meanNN, sdNN—reflecting parasympathetic and sympathetic functions—, RMSSD—representing the parasympathetic nervous system function—, and SHANNON entropy as a measure of complexity were calculated.

Within the frequency domain (FD), power spectra were calculated by applying fast Fourier transform with a Blackman Harris window on equidistant NN interval time series. To obtain equidistant time series, the tachograms were linearly interpolated and resampled at 10 Hz. From power spectra, the LF/HF ratio as index of the autonomic balance was calculated [28].

#### 3.4.2 Nonlinear dynamics methods (NLD)

*Symbolic dynamics (SD)*. Symbolic dynamics is a nonlinear method that describes the global short- and long-term dynamics of beat-to-beat variability on the basis of symbolization. Methods of nonlinear symbolic dynamics analysis have been shown to be suitable for the investigation of complex systems and describe dynamic aspects within time series. The method applied in this study (there are different approaches for deriving symbolic dynamics from a time series) is described in detail in [29]. In short, to classify dynamic changes within time series the heart rate time series were transformed into an alphabet of two symbols (“0” and “1”). NN intervals with a difference of under 20 ms were assigned a symbol of “0” and NN interval differences of over 20 ms were assigned a symbol of “1”. Based on the resulting series of symbols, words consisting of six characters were formed and then the index phvar20 quantifying high variability patterns (word type “111111”) was calculated.

*Poincaré plot analysis (PPA)*. The traditional PPA is a nonlinear quantitative technique of phase space characterization of time series fluctuations [30]. Each BBI is plotted as a function of the previous one. The shape of the cloud of points can be categorized into functional classes. Two linear indices, suggested by [31], describing the cloud of points in the Poincaré plot were calculated: short-term standard deviation in the perpendicular direction SD1 (minor axis) and long-term standard deviation along the longitudinal axis SD2 (major axis). SD1 and RMSSD are linearly interdependent (correlation of 1) because SD1 can be calculated using the equation $SD1=RMSSD/\sqrt{2}$ [32]. To avoid redundancy, SD1 was not considered separately in the following.

*Segmented PPA (SPPA)*. In contrast to the above mentioned indices from traditional PPA that describe only linear properties of time series, indices from the enhanced SPPA retain nonlinear features from time series [33]. For SPPA calculation, the cloud of points of the Poincaré plot will be rotated 45 degrees clockwise around their main focus and subsequently segmented into 12x12 equal rectangles. The size of the rectangles depends on SD1 and SD2. Finally, by means of occurrence probabilities of points in each cell of the 12x12 matrix, the complexity measure SPPA_6r [%] was estimated quantifying how many points are concentrated close to the point cloud center.

*Asymmetry analysis*. Asymmetry analysis, also known as irreversibility analysis, allows the detection of nonlinear irreversible dynamics in a Poincaré plot [34]. Here the distances between the individual points are translated into an identity line (diagonal). One representative of asymmetry analysis is the Guzik’s index [%] that quantifies the asymmetry of the HRV system based on the cumulative distance of each point to the line of the identity [35].

*Permutation entropy (PE)*. The permutations entropy is a complexity measure for time series based on analysis of permutation patterns [36]. For the calculation of permutation entropy, first, for each n consecutive NN intervals (n corresponds to the chosen embedded dimension) the rank order will be determined and stored as a symbol sequence. Secondly, the relative frequencies of all possible patterns of symbol sequences (permutations) related to the total number of symbol sequences will be estimated. Finally, permutation entropy is determined as the Shannon entropy of the distribution of pattern probability distribution. Here the standardized permutations entropy index normPE_embedd_3_lag_1 [bit] encoding three successive NN intervals (embedding dimension = 3 und delay time (lag) = 1) was determined.

A list and explanation of all parameters applied in this study is to be found in Table 2.

**Table 2:** List of applied HRV analysis methods and related calculated indices used in this study.

Method type	Analysis method	Index [unit]	Description
Linear	Time domain (TD)	meanNN [ms]	Mean value of NN interval time series
		sdNN [ms]	Standard deviation of NN interval time series
		RMSSD [ms]	Square root of the mean squared differences of successive NN intervals
		SHANNON [bit]	Shannon entropy of the NN interval density distribution (class width of 8ms)
	Frequency domain (FD)	LF/HF	Ratio of LF and HF, with LF and HF being the power in the “low” frequency range (0.04–0.15 Hz) and high frequency range (0.15–0.4 Hz)
Nonlinear	Symbolic dynamics (SD)	phvar20	Probability of occurrence of the word type “111111” reflecting high-variability patterns in the NN interval time series with NN interval differences>20ms over six heart cycles
	Poincaré plot analysis (PPA)	SD2	Standard deviation of the long-term NN interval variability
	Segmented Poincaré analysis (SPPA)	SPPA_6r [%]	Sixth row of a 12x12 probability distribution matrix determined on the basis of a 45 degree rotated and segmented Poincaré plot point cloud
	Asymmetry analysis	Guzik’s index [%]	Percent value of the sum of distances of points above the identity line in the Poincaré plot and the sum of distances of all points from the identity line
	Permutation entropy (PE)	normPE_embedd_3_lag_1 [bit]	Permutations entropy index encoding 3 successive NN intervals (embedding dimension = 3 und delay time (lag) = 1)

## 4. Statistics

Statistical analysis was performed with IBM SPSS Statistics 21 for Windows. Within the framework of descriptive statistics, mean values, standard deviations, median values and interquartile ranges for HRV indices were calculated for the yoga and control group separately for T1 and T2. Normal distribution and homogeneity of variance were checked applying the Shapiro-Wilk test and Levene test (both p>0.05). After visual inspection of histograms with normal distribution and quantile-quantile plots, non-normally distributed parameters were transformed into a normal distribution by log (indices: sdNN, RMSSD and LF/HF) and square-root (indices: phvar20 and SD2) transformation. Mixed 2×2 analyses of variance (ANOVA) were performed evaluating the dependent measures (HRV indices) for differences according to the measurement time (within-subjects factor: T1 vs. T2) or subject group (between-subjects factor: yoga vs. control), and subject group by measurement time interactions. To examine the effects of intervention (effects of the within-subjects factor: T1 vs. T2) on HRV indices, the yoga group and control group were separately analyzed in post-hoc tests using paired t-tests. For the analysis of group effects (effects of the between-subjects factor: yoga vs. control) on HRV indices at separate measurement times, unpaired t-tests were used as post hoc tests. Statistical significance was conducted at the significance level of p<0.05 to reveal significant effects of the intervention. The practical relevance of the main effects and interaction effects achieved by ANOVA was assessed by calculating the effect size partial omega-squared (ω_p_^2^, corresponds here completely to the lower case letter Omega with circumflex accent typically used) according to the calculation rules in [37, 38]. In contrast to the partial eta square which “consistently overestimates the true population effect size”, ω_p_^2^ has much less bias and “has been strongly recommended in most detailed, statistical discussions” [39]. Usually the effect strength is subdivided into small (0.01< = ω_p_^2^<0.06), medium (0.06< = ω_p_^2^<0.14) and large (ω_p_^2^> = 0.14) ones according to Cohen [40]. Some researchers [41] set negative values of ω_p_^2^<0 to the value 0 (no effect) which is factually correct, while other authors [42] have doubts in the sense of future meta-analyses and rather suggest to report such negative values as well (as in this paper), otherwise a systematic increase of the effects could occur. The effect size for t-tests was quantified by calculating the corrected, unbiased Hedges’ g* according to [43] with values of 0.2< = |g*|<0.5, 0.5< = |g*|<0.8, and |g*|> = 0.8 indicating small, medium, and large effects [37].

## 5. Results

The 20-min HRV data from 25 healthy young adults were evaluated prior to and following ten weeks of yoga (n = 14) or regular school sports as control (n = 11). Results regarding interactions, main effects and multiple pairwise comparisons from ANOVA and post-hoc t-tests are presented below and summarized in Table 3.

**Table 3:** Results of ANOVA and paired t-test quantified by descriptive statistics, significance values and effect sizes measures. To evaluate differences of nocturnal short-term HRV indices between a yoga group and a control group before and after a 10-week intervention.

	*Yoga (n = 14)*		*Control (n = 11)*		*GME*	*TME*	*IE*
	T1	T2	T1	T2			
*Parameter^1^*	M±SD	M±SD	M±SD	M±SD	p-value		
		g*-value		g*-value	ω_p_^2^-value		
*meanNN*	946.0±171.6	996.0±150.8	925.1±104.7	991.5±167.4	0.822	**0.033**	0.751
		0.301		0.458	-0.039	0.142	-0.037
*sdNN*	40.5±14.0	54.1±27.4 **#**	41.2±11.0	47.5±24.3	0.823	**0.037**	0.308
		0.544		0.234	-0.039	0.135	0.003
*RMSSD*	46.1±21.6	62.1±39.2 **#**	41.1±19.0	52.6±35.3	0.528	**0.024**	0.923
		0.489		0.388	-0.024	0.161	-0.041
*Shannon*	4.3 ±0.6	4.6±0.7 **#**	4.3±0.4	4.4±0.6	0.775	**0.036**	0.369
		0.539		0.266	-0.038	0.136	-0.007
*LF/HF*	0.86±0.49	0.80±0.46	1.60±0.93	1.05±0.78 **#**	0.158	**0.008**	0.094
		-0.263		-0.669	0.043	0.228	0.076
*phvar20*	0.11±0.11	0.19±0.17 **#**	0.12±0.14	0.15±0.19	0.760	**0.045**	0.713
		0.402		0.267	-0.038	0.123	-0.036
*SD2*	46.6±14.1	62.0±28.4 **#**	50.0±10.8	55.5±24.9	0.909	**0.039**	0.234
		0.644		0.213	-0.041	0.133	0.019
*SPPA_6r*	36.3±5.0	33.4±3.2 **#**	37.7±2.6	35.5±5.4	0.225	**0.010**	0.680
		-0.687		-0.493	0.022	0.217	-0.034
*Guzik‘s index*	48.0±4.4	51.2±4.2 **#**	47.8±1.6	48.8±4.7	0.324	**0.030**	0.237
		0.716		0.274	0.001	0.149	0.019
*normPE*	0.96±0.02	0.98±0.02 **#**	0.97±0.02	0.97±0.03	0.868	0.067	0.097
		0.579		0.028	-0.040	0.098	0.074

Values are expressed as mean value ± standard deviation (M±SD) at time point T1 (baseline before intervention) and T2 (post-intervention).

Abbreviations: ^1^all parameters and their units are explained in the methods section; n—number of participants; GME—group main effect; TME—time main effect; IE—interaction effect; normPE—normPE_embedd_3_ lag_1.

Effect size results quantified by: partial Omega squared ω_p_^2^-values in ANOVA with S—small effect with 0.01< = ω_p_^2^<0.06; M—medium effect with 0.06< = ω_p_^2^<0.14; L—Large effect with ω_p_^2^> = 0.14 and unbiased Hedges’ g*-values in t-tests with S—small effect with 0.2< = |g*|<0.5; M—medium effect with 0.5< = |g*|<0.8; L—Large effect with |g*|> = 0.8.

Significant results: p-values in bold indicating indices that had a significance level of p<0.05 for the tests GME, TME and IE and bold marked symbols # indicate also a significance level of p<0.05 for the paired t-tests (separately for yoga and control) of the time-dependent changes (T1→T2) in indices.

The statistical analysis of the interaction effects did not reveal a significant group and time interaction for the individual nocturnal HRV indices (no significant value of p<0.05 in column IE of Table 3). This observation could be confirmed by the one-way ANOVA, which showed that the HRV indices between the groups also did not differ significantly (small effects only) with regard to the individual nights (T1 and T2) (no significant differences between yoga and control related to the individual measuring times in Table 3).

Regarding the main effects for time regardless of the group (column TME of Table 3), all indices except normPE_ embedd_3_ lag_1 revealed medium and large effects (ω_p_^2^> = 0.06) achieving following significant values: meanNN [F_(1, 23)_ = 5.154; p = 0.033; ω_p_^2^ = 0.142], sdNN [F_(1, 23)_ = 4.894; p = 0.037; ω_p_^2^ = 0.135], RMSSD [F_(1, 23)_ = 5.803; p = 0.024; ω_p_^2^ = 0.161], Shannon [F_(1, 23)_ = 4.941; p = 0.036; ω_p_^2^ = 0.136], LF/HF [F_(1, 23)_ = 8.383; p = 0.008; ω_p_^2^ = 0.228], phvar20 [F_(1, 23)_ = 4.500; p = 0.045; ω_p_^2^ = 0.123], SD2 [F_(1, 23)_ = 4.818; p = 0.039; ω_p_^2^ = 0.133], SPPA_6r [F_(1, 23)_ = 7.918; p = 0.010; ω_p_^2^ = 0.217], and Guzik‘s index [F_(1, 23)_ = 5.361; p = 0.030; ω_p_^2^ = 0.149]. In detail, when comparing the values of the HRV indices before and after the intervention (T1 and T2) relatively clear differences were found. The changes in the HRV indices following the intervention were more dramatic for the yoga group than for the control group which is reflected in predominantly higher significances and stronger effect sizes in the yoga group (see Table 3, yoga: # in column 3, control: # in column 5).

In the yoga group significant improvements in the linear time domain HRV indices are evident: the values of SDNN [F_(1, 13)_ = 7.917; p = 0.015; g* = 0.544], RMSSD [F_(1, 13)_ = 5.350; p = 0.038; g* = 0.489] and SHANNON [F_(1, 13)_ = 7.815; p = 0.015; g* = 0.539] increased significantly after the yoga intervention. In both groups, meanNN values were higher (representing a slower heart rate) in the second measurement than in the first one, though the differences were not significant (yoga: p = 0.166; controls: p = 0.114) and the effects only small (yoga: g* = 0.301; controls: g* = 0.458).

When looking at the linear frequency domain, for LF/HF, only a slight trend with small effect size towards a decreasing ratio value after yoga intervention could be identified (p = 0.383, g* = -0.263). For the control group, a medium effect for a significant reduction of the LF/HF ratio [F_(1, 10)_ = 8.732; p = 0.014; g* = 0.669] after ten weeks of regular school sports was noticed.

Considering the nonlinear dynamics methods, from Poincaré plot analysis (PPA), the linear SD2 index [F_(1, 13)_ = 7.499; p = 0.017; g* = 0.644] was significantly increased after yoga intervention than before yoga. The nonlinear index SPPA_6r [F_(1, 13)_ = 5.945; p = 0.030; g* = 0.687] of the segmented PPA decreased significantly following the yoga intervention. Furthermore, the indices phvar20 [F_(1, 13)_ = 4.831; p = 0.047; g* = 0.402] of symbolic dynamics, Guzik’s index [F_(1, 13)_ = 6.880; p = 0.021; g* = 0.716] of asymmetry analysis and normPE_embedd_3_lag_1 [F_(1, 13)_ = 10.217; p = 0.007; g* = 0.579] of permutation entropy analysis increased significantly from the first to the second measurements for the yoga group. With regard to the control group, significant changes in nonlinear HRV indices comparing T1 and T2 could not be identified.

## 6. Discussion

In our physiological analysis of the effects of Yoga in schools on young adults, a supposed shift towards improved autonomic regulation after the 10-week yoga course could be recognized in the yoga group. Referring to the within group comparisons (Table 3), higher effect size values and more significant increases in the HRV indices sdNN, Shannon entropy, and SD2 from PPA show a rise of overall HRV, greater HRV complexity and unpredictability (increasing entropy) after yoga intervention [28, 25, 24].

This is made even clearer by certain nonlinear HRV indices. Both, increased values of normPE_embedd_3_lag_1 from permutation entropy and phvar20 from SD and a decrease of sppa_6 from SPPA suggest more complexity, unpredictability and variability of NN interval time series gained after yoga intervention in comparison to time series acquired prior to interventions and after regular school sports [44, 45]. A noticeably higher value of phvar20 in the yoga group after intervention means an increased occurrence of words of the word type “111111” within the NN interval time series indicating increased percentage of patterns with higher variability (>20 ms). Compared to the baseline, the lower sppa_6 value following the yoga course describes a redistribution of points near the main focus of the point cloud of the Poincaré plot to outer parts of the cloud confirming also a higher HRV and greater complexity. In the control group, no such changes in the HRV indices could be observed, only minor effects of regular school sports on the nonlinear indices were found. In addition, in accordance with [37], the values of the individual effect sizes of the nonlinear indices for controls were noticeable smaller (mainly small effects) compared to the yoga group (predominantly medium effects).

Referring to the autonomic nervous system, an increased parasympathetic nervous system activity following the yoga course can be interpreted from the increases in RMSSD and Guzik’s index. The Guzik’s index values increased from a baseline value of 48% to over 51% following the yoga intervention indicating a shift in heart rate asymmetry from acceleration to deceleration. Regarding the Poincaré plot, after yoga intervention, the points that lie above the identity line are further away than the points lying below it reflecting an asymmetric oscillation that enhances NN interval prolongation and inhibits shortening and thus an increase in vagal tone [46, 35]. Here, too, changes of RMSDD and Guzik’s index were not found in the control group.

In general, our explorative pilot trial demonstrated an improvement of HRV (more parasympathetic dominance and overall higher HRV) after yoga interventions compared to regular school sports, assuming an improved self-regulation of the autonomic nervous system as a result of the yoga course. This is justified by the considerable changes in the within group comparisons of the yoga group. A direct comparison of the individual HRV indices showed both larger effect sizes and smaller significance sizes for the yoga group compared to the control group for the majority of the indices (8 out of 10). This indicates more pronounced changes in HRV in the yoga group.

Interactions between groups (yoga and control) and time (T1, T2) and also group main effects could not be found. No discernible changes in the between group comparisons at T2 occurred. Looking at the ranges of most index values for T1 and T2, it is noticeable that the controls are narrower and within the ranges of the yoga group. This is very likely to result in ANOVA not being able to detect any main group as well as interaction effects. In addition, the HRV data of the participants at the beginning of the intervention at T1 were distributed very different (as it is normal with HRV data, because it depends on many various factors). This is why the outcomes at T2 were also at different levels. They changed in the direction of an improved HRV in the yoga group, but because the effects are not very strong, the baseline data are very individual and the investigated group sizes are very small, the changes in the between group comparisons demonstrate no significances.

In studies investigating HRV in relation to cerebral blood flow, it was shown that there are connections between HRV and areas of the brain responsible for physical stress reactions. In this sense, these results support the hypothesis that an increase in HRV is associated with an improved ability on the part of the organism to adapt to exterior influences and overall greater well-being [47]. Students who experience less stress due to specific stress-reducing measures demonstrate a greater variability in heart rate [48]. The results of the study presented here corroborate these effects and correspond to the results of the study’s psychometric analysis where a significant reduction of the Cohen Perceived Stress Scale was found in yoga participants in comparison to the control group [23].

In teaching students how they can improve dealing with stress and reduce the feeling of stress, learning behaviors can be optimized—for example as a result of better concentration, emotional resilience and alertness—and the negative long-term effects of stress can be avoided. All these aspects have been demonstrated in studies on yoga in school sports utilizing psychometric data [11, 18].

In regards to HRV frequency domain indices, this study did not yield such clear results. Based on the mean LF/HF values of the first measurement T1 it is evident that on average the HRV in the control group was influenced to a greater extent by the sympathetic nervous system than the HRV in the yoga group. The difference of LF/HF between T1 and the second measurement T2 is thus noticeably large for the control group, while for the yoga group only a slight trend is evident, whereat the LF/HF values of both the control and intervention groups shifted towards parasympathetic dominance. Why the frequency parameter LF/HF of the control group demonstrates such a high activation of the sympathetic nervous system during the first measurement can only be speculated. Possibly, some subjects of the control group were coincidentally more agitated or excited at T1 than T2 which could explain the wide LF/HF interquartile range of 0.67–2.44 at T1 in contrast to the LF/HF range of 0.38–1.51 at T2. That the statistical test at time T1 did not reveal a clear difference for LF/HF in comparison between the control and future yoga participants may be a result of the distribution of the data in the small test groups (11 controls, 14 yoga participants).

One strength of the study is the use of a control group. The intervention and control groups are comparable insofar as the yoga course was held at the same place and time as the standard sports class and that the sports class for the students in the control group was not changed. The professionalism of the yoga teachers (BDY certification) and the standardization of the intervention should also be emphasized. Together, the yoga teachers created a catalogue of exercises to provide orientation for the individual yoga sessions. To ensure the best possible comparability of HRV data, the 20-minute undisturbed sections of the long-term ECGs that were analyzed were taken manually at similar times (during night sleep) and were carefully preprocessed in the same way. In addition, a broad spectrum of HRV indices were used, not only from linear time and frequency domain (which are used far more frequently) but also from the analysis of nonlinear dynamics which additionally can reveal and characterize the dynamics and complexity of nonlinear systems [24].

Among the study’s limitations are the fact that the subjects were not randomized, which was not possible because the study was conducted in the framework of fixed school routines. Each of the three sports teachers assigned one of their classes to the yoga group and one to the control group. Though the comparability of the students was given priority it cannot be entirely ruled out that subjective sensitivities and emotional constitutions played a certain role. Of decisive importance is the fact that there were no noteworthy differences in the HRV indices in the baseline of the intervention and control groups. The number of 25 students is small, for which reason only forward-looking but no precise general interpretations relative to the overall population are possible. However, we consider—precisely because of the small number of subjects—the effects on the population in question to be quite notable. A further limitation due to the group size/ number of parameters is the possible increase of the alpha error. This can be accepted insofar as it is an explorative study. Interestingly, there is no general accepted effect size estimator, each measure has its own characteristics and there are many difficulties in interpretation. In this paper, the Cohen [40] and Lakens [37] classifications of effect sizes were used as a general rule of thumb.

Unfortunately, we could only investigate the differences in HRV changes between controls and a yoga group using BBI time series of the night phases. BBI time series of wakefulness would more directly represent the use of yoga for stress management and related changes in HRV. However, it was not possible for the majority of students to find suitable and comparable undisturbed 20-minute BBI day segments (early, afternoon, evening) because the individual daily activities of the students caused various artefacts and disturbances in the ECG. The exclusion or correction of such frequently occurring disturbances would have led to more or less dramatic falsifications of the BBI time series and thus to wrong results and interpretations. For this reason, the evaluation of BBI time series of the daily phases had to be discarded. Though such factors as illness, pregnancy and mobility limitations were excluded by inclusion and exclusion criteria defined for the study, it cannot be entirely ruled out that other factors affected the HRV parameters (for example minor infections or physical or emotional strain). It was not possible in the framework of the study to check for such factors. Additionally, investigations about effects of age and gender on the outcome of yoga intervention particularly on the HRV indices were not possible due to small participant groups and the selected collective of participants (only students aged 17–25 years of secondary schools). The length of the yoga intervention of ten weeks is to be considered quite short. In addition, some of the students did not attend every session, which also limits the effectiveness of the intervention. In both groups, the participants took part in almost eight from ten lessons (yoga group: 55.86%; control group: 77.27%). In the yoga group, four young adults did attend every session, while in the control group no participant did.

It would had been good as well to measure the home practice of the yoga students and include it in the analysis. Taking into account the issues mentioned in the limitations, in subsequent studies the presented results need to be validated by analyzing a larger amount of yoga participants.

## 7. Conclusion

In conclusion, the HRV data confirm the impression made by the evaluation of the psychometric data. Here it was demonstrated that yoga in the framework of school sports is an effective measure to reduce stress perception and the accompanying psychological symptoms, for example anxiety and depression. This finding is corroborated by modifications in the autonomic regulation of HRV, which was considerably improved (more complexity, unpredictability and variability of beat-to-beat time series) after the 10-week yoga course. Further studies should be conducted that include randomization, long-term follow-up measures and larger sample sizes.

## Supporting information

S1 File (PDF)

S1 Data (XLSX)

S1 Raw data (ZIP)
