# Supplementary material for: Yoga in school sports improves functioning of autonomic nervous system in young adults: A non-randomized controlled pilot study
Source: PLoS One. 2020 Apr 13;15(4):e0231299. doi: 10.1371/journal.pone.0231299 (PMC7153865; doi:10.1371/journal.pone.0231299)
Supplement: S1 File — (PDF) [file pone.0231299.s001.pdf]

Request for advice from the Ethics Committee to conduct a medical-scientific project, which includes neither the clinical trial of a drug or medical device

1. Title of the study	"Yoga in school sports, "Non-randomized controlled pilot study to evaluate the effects of school stressredukction Yoga for students * inside of the Ber-lin school centers (OSZ)
2. Ethics Commission's application number	*EA2 / 023/16*
3. Decisions of other ethics committees in the same case	
4th The study and its objectives; Indication of the hypotheses, separated into primary and secondary hypotheses and clinical parame-ters (primary and secondary endpoints), based on which the hypotheses are tested	The main objective is the scientific monitoring and evaluation of the effects of a 10-week yoga classes as an innovative standard part of OSC physical education on feelings of stress, quality of life and mental perception in Berlin OSZ students * inside compared to a control group that the regular OSZ physical education visited.<br><br>Hypothesis: A 10-week yoga classes leads to a reduction of the stress strength and has a positive effect on mental and physical sense as well as the concentration of.<br><br>**MAIN OUTCOME (not confirmatory):**<br>o Comparison of the groups in the course with regard to the stress intensity using the Cohen Perceived Stress Scale (CPSS)<br><br>**Secondary outcome parameters:**<br>o WHO-5 (Quality of Life) questionnaire<br>o Questionnaire on depression and anxiety (HADS) and mood (POMS)<br>o Visual analog scales for the following symptoms: general pain, headaches, shoulder and neck tension, fatigue, sleep quality<br>o Questionnaire on the concentration (Test d2-R)<br>o Questionnaire on mindfulness (Mindful Attention and Awareness Scale)<br>o Questionnaire for the effectiveness of the practice of yoga (yoga Self-Efficacy Scale)<br>o Overall assessment of the efficacy and tolerability (Likert scale) on the part of volunteers<br>o Compatibility based on the query of adverse events<br>o Measurement of the stress response / changes in the autonomic nervous system, autonomic means 24 ECG<br><br>In addition, a qualitative detection as part of focus group interviews, how does yoga of OSZ students * inside, used and evaluated.
5. Explanation of the importance of the study	So far no data exists about the extent to which yoga can in the school context conducive affect alertness, concentration, resiliance and general quality of life. The study will contribute to clarify the role yoga can play in German schools as an element of the regular physical education.

6. Which of the following provisions shall apply<br>a) Medical Devices Act In accordance with § 23 b MPG - except the klin. exam<br><br>b) Radiation Protection Ordinance § 23<br>c) Röntgenverordnung § 28a<br>d) Gendiagnostikgesetz<br>e) Privacy laws:<br>- Specific indication of by the to be met responsible entity Data Protection Act (= for the Charité Berlin Data Protection Act - BlnDSG).<br><br>- Possibly. protection laws corresponding to the group of participants, in addition to be observed Landesdaten- or BDSG.	Privacy laws
7. If necessary, .: Description and characterization of the test products	----
8. essential results of pre-tests or reasons for not implementing the same	-
9. Gist and results of previous studies / applications in the study products to be tested	Yoga has been shown in adults in a variety of studies to be effective in improving mental and physical health and in the reduction of stress. Some studies point to the potential stress-reducing, mentally balancing effects of yoga in adolescents.<br>In a controlled randomized companion study (n = 136) was already stress-reducing effect of yoga are described in adolescents (Khalsa 2012). A non-randomized controlled trial showed a significant decrease in salivary cortisol in the yoga group compared to the control group (West 2004) in adolescents in addition to stress-reducing effects.<br>Recent review articles on Yoga in adolescents emphasize the protective and preventive importance of yoga in the maintenance and improvement of mental and physical health (Hagen 2014 Galatino 2008 Birdee 2009).
10. Description of the proposed measures / methods of investigation and any deviations from the in the med. Practice usual measures / investigations (What is "routine", which is conditional deviation in the study?)	There are exclusively non-invasive surveys using internationally validated questionnaires in German.<br><br>how Yoga is personally assessed and evaluated in addition is requested after 5 and 10 weeks at 15 students in the yoga group as part of 45-minute group interview. Which participated in the group interviews pupils * get in as a thank you a bookshelf, a cinema voucher. The offer is made to participate in the interviews for selection before randomization. The focus group interviews are recorded for the qualitative detection in a phonogram and then transcribed and content analysis.<br>takes place at 40 subjects by selecting according randomization the inclusion of a 24 Holter ECG to measure the stress response before the start, after 10

	weeks of intervention and 6 months after completion of the study.<br><br><u>ethical aspects</u><br>The study will be conducted in accordance with international guidelines for clinical trials (Declaration of Helsinki, ICH-GCP) and the research ethical framework of sociological accompanying research.
11. Review and consideration of the foreseeable risks and inconveniences of study participation compared with the expected benefits for the participants and future ill persons (benefit-risk assessment)	A simple program of yoga was used, so there is no greater risk of injury than conventional school sports is expected. The intervention is not used primarily for the study, but already then re-inserted, but regular part of physical education in the OSZs. The results can contribute generally to an improved evaluation of the benefits of school sports.<br><br>The inclusion of a 24 Holter has no disadvantages for the subjects.<br><br>In summary, there is a positive benefit-risk assessment.
a. to be tested medical benefits for study participants(Individual benefits for individual patients)	Participants in both groups will benefit from a potentially active effect through yoga. The yoga group receives yoga classes as part of the school physical education. Benchmarks can include termination of the observation period, free to participate in a content and timing identical yoga class outside of regular school hours at selected Berlin yoga centers (voucher). This results in a foreseeable potential benefits through participation in the study for all subjects.
b. to be tested medical benefits for future ill persons(Group benefits)	Yoga will prove in the classroom as helpful in maintaining and improving the mental and physical sense, they could yoga exercises in the future in schools applied and benefit other students.
c. Risks and burdens to research participants(List All in detail)	All surveys are non-invasive.<br>Yoga has no greater risk than conventional school sport and is a safe exercise method in accordance of guided practice (Cramer 2013). Overall there is no serious public health risk from study participation.
12. risk control measures	Participants are encouraged any possible adverse events (AEs) to be reported immediately. UE are evaluated by the investigator and investigators and monitored according to the study progress.
13 termination criteria	Each UE is judged by the investigator as to its severity and the possible link with the Yoga program. Serious AEs and adverse events must be reported by the investigator within 24 hours by phone, fax or telegram to the head of the clinical trial.<br>Takes the subject's consent back, so the investigator should discontinue the study for him. This is noted on the report cards.<br>The study can be aborted if it is evident that the study can not meet these requirements. Which includes:<br>-It occur serious protocol violations<br>the report forms to be filled out poorly or fraudulently<br>-legally or ethical rules are not respected<br>-An early departure for reasons mentioned above, is only possible in agreement between investigator and investigators.<br><br>If serious UE or serious northwest on or piling up non-

	serious AEs, the investigator may stop the study in its sole discretion.
14. Number, age and sex of the persons concerned	120 students from Berlin OSZ centers from 17 to 27 years.
15. Biometric planning, indicating the statistical methodology, including the reasons for the falling number.<br>Specify the / the statistician / Statistician	This is a descriptive and exploratory analysis without statistical sample size calculation in a non-randomized pilot study.<br><br>The following sample sizes are set:<br><br><u>patient numbers</u><br>• Screening patients (n ≈ 120)<br>• included in the study, patients (n = 110) - intention to treat<br>• Patients whose data is subjected to statistical analysis (n = 100) - Per Protocol<br><br><u>hypotheses</u>:<br>H0: Efficacy of yoga classes in stress reduction = effectiveness of the regular physical education in stress reduction<br>HA: Efficacy of yoga classes in stress reduction ≠ effectiveness of the regular physical education in stress reduction<br><br><u>data analysis</u><br>The primary, not confirmatory, endpoint is the group difference in the course of Cohen Perceived stress scores after 10 weeks.<br><br>The above secondary objective parameters are collected at the beginning (baseline), after 10 weeks and after a total of six months.<br><br>The baseline is applicable to two times (before and after the Easter holidays) to account for any possible confounders. The second baseline value is used as a covariate in the analysis of covariance.<br><br>The statistical analysis of the study is carried out at the Institute of Epidemiology, Social Medicine and Health Economics, Dep. Naturopathy by Mr. Dipl.-Biol. Nico Steckhan.<br>All adverse events occurring are described individually.<br><br><u>Heart rate variability analysis:</u><br>The sample size of 40 subjects meets the requirements of the heart rate variability analysis. The long-term ECGs are Prof. Dr. Michalsen (Specialist in Cardiology) examined. In a corresponding abnormalities further diagnosis will be made.
16th<br>a. Statement and possibly explaining the inclusion and exclusion criteria	**<u>Inclusion criteria:</u>**<br>○ Students from Berlin school centers (OSZ) aged 17-27 years<br>○ Written consent of students (and their parents (with minority 17 years)

	**<u>exclusion criteria</u>:**<br>○ Severe chronic or acute diseases<br>○ Pregnancy or breastfeeding<br>○ Immobility or limitation for gymnastic exercises orthopedic, neurological or other medical causes<br>○ Participation in another study
b. Study information (who those provided verbally and in writing, stating how much time between informed consent remains (written information as an attachment)	- Investigators enter personal / oral the first information<br>- intermediate information and consent to participate remain at least 24 h
c. Consent (written form as an attachment)	s. investment
d. Possibly. Informed consent of the legal representative (if necessary, also the descrip-tion of the method for establishing a court care)	Written consent of the parents of students in minority
17. measures for the recovery of study partic-ipants (notice?, Newspaper advertisements? Etc.)	Consultation with Berlin OSZ schools offering yoga as part of physical education curriculum-compliant written consent of participating students (or their parents also in minority).
18. If necessary, .: reason for including and explanation of the therapeutic benefit for people who are underage and / or not capa-ble of giving consent.	Prolonged stress damaging for young people to health behavior can already cause (see above). There have already been positive evidence for stress reduction and improvement of mental and physical health in adolescents.
19 relationship between study participants and study physician / -ärztin (If the study doctor at the same time, the attending physician?)	No
20. Declaration on the inclusion of possibly dependent on the sponsor persons	-
21. Measures that allow for determining whether a study participant in several studies at the same time or before the expiry of a specified period in the previous study teilnimmt.Ist participating in several studies possible?	All subjects are explicitly asked for an otherwise participate in the study and noted that a simultaneous participation in other clinical trials throughout the study period is not possible.
22. If necessary, .: payment or reimbursement of the study participants (height, what is to be paid?)	---
23. If necessary, .: plan for further treatment and medical care of the persons concerned after the end of the study	---
24. If necessary, .: Insurance of the study participants (confirmation of insurance and insurance conditions, insurers, insurance coverage, insurance duration)	As part of the school physical education students are * inside insured through the Unfallkasse their school.

25. Documentation procedures:<br>- Possibly. Reference to the CRF arches.<br>- sample handling<br>- Storage / archiving (incl. Deadlines)<br>- Access to the data and samples	CRF are created.
26. If necessary, .: description of how the health of healthy people affected should be documented	----
To document 27. If necessary, .: Methods to determine adverse events and inform (when, by whom and how ??)	Initial message is from study participants to investigators or investigator. This will be reported in writing within 24 hours to the study director and documented in writing.
28 action to safeguard the confidentiality of stored data, documents and, if necessary, samples, demonstration of pseudonymous or anonymous data and samples from study participants **(Initials and date of birth as encoding scheme are not allowed!)**<br>- description of the separation of medical records, study documentation and assignment of the personal data<br><br>- Identification of the access rights, including access to Teilnehmeridentifikations- list during and after the execution Studiendurch-<br><br>- Detailed specification of the procedures for transfer, encryption, blocking and deletion (including specifying the network structure and used server optionally used).<br><br>-possibly. Access to identifying data for legally authorized auditor (third parties) to<br>earmarked inspect the<br>for needed files.	In the present study, all subjects data in the report forms and the database are identified only by a number subjects. The list of personal data is kept separate from the database and remains within the Charité and the Immanuel Hospital.
29. Declaration on compliance with data protection<br>- assurance that all the information collected about the subscriber study and stored confidential (secret data and patient confidentiality) to be treated.<br><br>- assurance that the identifying<br>Data is only accessible to the investigator or authorized by him employees.<br><br>- the measures to ensure confidentiality	According to the Federal Data Protection Act (Act) § 4 (1).<br>All recovered audio data will only password-protected with the program Winzip to an external media stored and will not be transferred to third parties. In addition, media are kept under lock and key with the appropriate files when not in use to protect against theft and damage. In the transcribed data are completely anonymous all information necessary to enable a conclusion to be volunteers.

- Measures to privacy-friendly way transmission of data that can be produced no personal reference to third parties.<br><br>- provide information indicating the opposition and unloading facilities,<br><br>- measures to ensure the rights of participants.	
30. Names and addresses of facilities that are integrated as a study center or laboratory study in the study, and the study director and investigators<br>- Detailed specification involved external Service with details of data access possibility.	Charité University Clinic for Naturopathy<br>at Immanuel Hospital<br>Königstr. 63<br>14109 Berlin<br>Tel. (030) - 80505-691<br>Fax (030) - 80505-692<br>E-mail: a.michalsen@immanuel.de<br>Head of study: Prof. Dr. A. Michalsen<br>Investigators: Dr. med. Michael Jeitler, Christoph Hohmann<br><br>The analysis of heart rate variability is done by:<br>Charité - Universitätsmedizin Berlin<br><br>Department of Pediatric Oncology and Hematology<br>August Platz 1<br>13353 Berlin<br>Tel. (030) - 450-666058 (Mrs. vom Hoff-Heise)<br>Fax (030) - 459-566930<br>E-mail: georg.seifert@charite.de<br>Prof. Dr. G. Seifert
31 representations about the suitability of the inspection body, in particular the adequacy of existing there resources and facilities and the time available to conduct the trial staff and experience in conducting similar studies	The establishment has several years of experience in conducting clinical trials and has already carried out numerous clinical studies. These studies were carried out successfully within the given schedule. At the facility experienced staff and adequate facilities and continuous usable modern PC workstations are available in studies.
32. Agreement on access of the investigator / principal investigator / principal investigator, to the data and the principles governing the publication.<br>- Publications in a form that allow no conclusion on the person.	The data are accessible only through the study leader. The publication takes place regardless of the result in a recognized journal.
33. Details of the financing of the study:<br>Funding source (name and seat) and amount of funding in €.	The study is financed by home remedies of Immanuel Hospital and donations of BDY (Association of German Yoga Teachers).

Name and signature of the applicant:

I hereby certify that the information provided in this application is correct. I am of the opinion that it is possible the study mentioned above in accordance with the Protocol to carry out the national legislation.

I understand that I am required under Section 19 Berlin Data Protection Act (BlnDSG) to create a file and process description for automated processing of personal and personal beziehbarer data and

§19a according must provide the DPO Charité available. I am informed that if there is a method used to process the data to professional secrecy (eg medical confidentiality) subject, I have to arrange for a prior checking by the DPO of the Charité §5 BlnDSG before using this method and I may use the process only for a positive test result.

Name: Prof. Dr. med. Michalsen

First name: Andreas

Address: King Street 63; 14109 Berlin

Position: Chief Physician, Professor of Clinical Naturopathy of the Charité

Date:
